# Emerging environmental health risks associated with the land application of biosolids: a scoping review

**DOI:** 10.1186/s12940-023-01008-4

**Published:** 2023-08-21

**Authors:** Elizabeth A. Pozzebon, Lars Seifert

**Affiliations:** California Conference of Directors of Environmental Health, P.O. Box 2017, Cameron Park, CA 95682-2017 USA

**Keywords:** Biosolids, Emerging pollutants, Land application, Microplastics, Organic contaminants, Per-and polyfluorinated alkyl substances (PFAS), Pharmaceutical and personal care products (PPCPs)

## Abstract

**Background:**

Over 40% of the six million dry metric tons of sewage sludge, often referred to as biosolids, produced annually in the United States is land applied. Biosolids serve as a sink for emerging pollutants which can be toxic and persist in the environment, yet their fate after land application and their impacts on human health have not been well studied. These gaps in our understanding are exacerbated by the absence of systematic monitoring programs and defined standards for human health protection.

**Methods:**

The purpose of this paper is to call critical attention to the knowledge gaps that currently exist regarding emerging pollutants in biosolids and to underscore the need for evidence-based testing standards and regulatory frameworks for human health protection when biosolids are land applied. A scoping review methodology was used to identify research conducted within the last decade, current regulatory standards, and government publications regarding emerging pollutants in land applied biosolids.

**Results:**

Current research indicates that persistent organic compounds, or emerging pollutants, found in pharmaceuticals and personal care products, microplastics, and per- and polyfluoroalkyl substances (PFAS) have the potential to contaminate ground and surface water, and the uptake of these substances from soil amended by the land application of biosolids can result in contamination of food sources. Advanced technologies to remove these contaminants from wastewater treatment plant influent, effluent, and biosolids destined for land application along with tools to detect and quantify emerging pollutants are critical for human health protection.

**Conclusions:**

To address these current risks, there needs to be a significant investment in ongoing research and infrastructure support for advancements in wastewater treatment; expanded manufacture and use of sustainable products; increased public communication of the risks associated with overuse of pharmaceuticals and plastics; and development and implementation of regulations that are protective of health and the environment.

**Supplementary Information:**

The online version contains supplementary material available at 10.1186/s12940-023-01008-4.

## Background

During wastewater treatment, solids are separated from liquids and are then treated physically and chemically to produce a semisolid, nutrient-rich product known as biosolids or sewage sludge. Biosolids are typically disposed of through landfilling, incineration, or are used as a soil amendment (fertilizer) as they contain high concentrations of nitrogen, phosphorous, organic carbon, and other essential elements which are beneficial for soil quality and crop production [1–3]. Although the benefit of recycling nutrients necessary for crop production and avoiding the use of energy-intensive synthetic fertilizers is significant, biosolids also act as a sink for emerging pollutants [3–21]. Preventing harmful exposures to these emerging pollutants when land applied remains a challenge [10, 20, 22]. The debate over safely using these human waste-derived biosolids as soil amendments is ongoing [23].

The US EPA standards for determining biosolids quality are found in Title 40 of the Code of Federal Regulations, Part 503, but are limited in focus to the presence of ten inorganic metals (As, Cd, Cu, Hg, Mo, Ni, Pb, Cr, Se, and Zn), pathogens, and vector attractiveness. These standards do not currently contain regulatory standards or thresholds that pertain to the presence of synthetic organic contaminants [10]. And, while many organic compounds degrade easily and have minimal harmful effects on the environment, other more toxic organic contaminants meet the US EPA’s definition of being persistent and can accumulate in environment, causing harm to humans and wildlife when land applied [24]. In addition to the lack of standards for monitoring persistent and toxic organic contaminants in biosolids prior to land application, there are significant gaps in our understanding of fate of these pollutants once land applied and the synergistic effects of multiple organic compounds on their distribution and transport within the environment. Moreover, the lack of efficient technologies to detect and measure these organic contaminants further reduces our ability to monitor their presence in the environment and evaluate potential impacts on human health.

The volume of biosolids produced in the US is not inconsequential. The US EPA estimates that, in states where they are the permitting authority, 4.5 million dry metric tons were produced in 2021 with nearly half (43%) being land applied [25]. The remaining biosolids were landfilled, incinerated, or managed by other methods such as storage or deep well injection. The US EPA also notes that the actual amount produced could be as much as 6 million dry metric tons according to a 2018 survey conducted by the North East Biosolids and Residuals Association, because it additionally accounts for states where US EPA is not the permitting authority. The global market for biosolids was estimated at 7.5 billion USD in 2022 and is projected to reach 10.7 billion USD by 2030 [26].

This scoping review provides a landscape of the current research regarding emerging pollutants in biosolids and their fate in the environment when land applied. Potential pathways of exposure, current detection methods, and possible impacts on human health and the environment are discussed. The need for additional research on the fate of these pollutants and their synergistic effects in the environment along with the significant need for novel treatment methods and detection technologies for emerging pollutants is highlighted. The authors call critical attention to the many knowledge gaps that currently exist to guide state and Federal regulatory frameworks for human health protection when biosolids are land applied.

## Methods

We used an iterative process to select academic and governmental publications for inclusion in this scoping review. Initially, we searched databases and governmental websites to identify publications associated with risks to human health and the environment from the land application of biosolids using the following terms combined with “biosolids” or “land application of biosolids”: *contaminants*; *organic contaminants*; *emerging pollutants*; *PFAS*; *microplastics*; *ground water*; *surface water*; *plant uptake*; *wildlife*; *agriculture*; *health risk*; *benefits of*; *pharmaceutical; personal care products*; *antibiotic*; *endocrine disrupter*; *treatment technology*; *diagnostic techniques; regulations*; *and fate and transport*. Publications were initially screened for relevance using the title and/or abstract, and those relevant to biosolids land application were reviewed in their entirety. Authors identified through the literature search were contacted for further discussion regarding their study findings and were asked for suggestions on additional publications or resources for inclusion. References from identified publications’ citation lists were also reviewed to identify other applicable resources. A concerted effort was made to establish an exhaustive list of studies that were published in the last decade (between 2011 and 2022) pertaining to the land application of biosolids.

## Results

A total of 172 scholarly research articles and governmental reports were included in this scoping review (Supplemental Fig. 1). Biosolids contain nutrients and energy which can be used in agriculture or waste-to-energy processes [11] or to replenish organic carbon in soils. However, while they do contain valuable nutrients (mostly nitrogen and phosphorus), they also contain a range of synthetic organic compounds. These organic compounds are produced for a variety of purposes, including healthcare, agriculture, and transportation, and are considered indispensable for modern society [27–29]. While many organic compounds degrade easily and have minimal harmful effects on the environment, other more persistent synthetic organic contaminants have the potential to accumulate in biological matrices and can eventually cause harm to humans, wildlife, and the environment [24, 30]. Conventional wastewater treatment plants were not designed to remove these emerging pollutants and currently they are only partially effective in removing or degrading synthetic organic compounds [22, 31] resulting in the accumulation of these pollutants in biosolids.

While the risk of direct human exposure to emerging pollutants in biosolids is low and realistically may involve only those who work with biosolids such as farmers and biosolids workers [32], the risk of indirect exposure is significantly higher. Not only can the land application of biosolids result in ingestion of contaminated food-crops, animal up-take in meat or milk, and drinking water contamination, but it can also lead to pollutant exposure via inhalation [14, 19, 32–36]. Although exposure to individual synthetic organic pollutants in biosolids such as antimicrobials, pharmaceuticals, personal care products, surfactants and hormones would not accumulate in the food chain at concentrations that may pose a risk for human health, the sum of them could be of considerable concern [11, 37].

In this review we focus on three broad classes of emerging pollutants that pose the most significant risks to human and environmental health when biosolids are land applied to agricultural soils. Then, we explore the fate, transport, and synergistic effects of these emerging pollutants generally in the environment. Finally, we discuss current diagnostic tools, treatment methods and the critical need for the development of standards to protect human and environmental health.

### Emerging pollutants

#### Microplastics

Microplastics, plastic pieces less than five millimeters in diameter, are typically the result of larger plastic debris degrading into smaller sizes; however some microplastics, such as microbeads, are manufactured at micro sizes and are often used in commercial health and beauty products [38]. Microplastics easily pass through wastewater treatment systems and approximately 70 to 98% of microplastics from liquid wastewater accumulate in biosolids during the treatment process [20, 39–41].

Unsurprisingly, the land application of biosolids has led to agricultural soils being one of the largest natural reservoirs of microplastics [20]. Corradini et al. [13] evaluated 31 agricultural fields and found that concentration of microplastics in agricultural soils increased over time after successive land applications of biosolids. Soils with 1, 2, 3, 4, and 5 applications of biosolids had medians of 1.1, 1.6, 1.7, 2.3, and 3.5 particles g^− 1^ dry soil, respectively, demonstrating that microplastic concentrations were significantly correlated with biosolids applications. In addition, microplastics from land applied biosolids do not always remain in the soil but can also be released into the surrounding environment [3, 5]. Although the true scale of microplastic contamination has yet to be assessed, studies have repeatedly detected microplastics at significant distances from their source of origin and at higher elevations, indicating their susceptibility to becoming airborne [3, 42–46]. Inhalation of microplastics is associated with oxidative stress in lung tissues, along with general inflammatory responses in airways and bronchi and chronic exposure can lead to death [3, 47, 48]. Several other studies have also directly measured microplastic concentrations suspended in air or dust, [49–52] deposited on land, [53, 54] or trapped on tree canopies [3, 55]. And because the impact of the horizontal and vertical intra- and inter-ecosystem spread of microplastics in the environment remains unquantified on the whole ecosystem [56], and ultimately on humans via the food chain, this area needs further investigation, particularly because microplastics are persistent in the environment and can accumulate in soil [3, 20, 57, 58].

#### Per- and polyfluorinated alkyl substances (PFAS)

Per- and Polyfluorinated Alkyl Substances (PFAS) is a broad term for manmade aliphatic compounds with at least one carbon-fluorine (C-F) bond [14, 59]. PFAS have been mass produced since the 1940s [14, 59]; however, due to environmental concerns, the production and use of long-chain (≥ 8 carbons) PFAS in North America, Europe, and Australia were voluntarily phased out in the early 2000s and replaced with shorter-chain PFAS [59, 60]. Short-chained PFAS have a lower tendency to be absorbed or leached into the soil and bioaccumulate, but are also more mobile in the environment than the longer chain (C8) compounds [19, 59, 61] increasing risks of groundwater contamination and human exposure. Short-chain PFAS replacements also still persist in the environment and can have adverse health and environmental effects [62–64].

A study measuring perfluoroalkyl carboxylic acid (PFCA), a subset of PFAS, in biosolids from multiple wastewater treatment plants in the US, Canada, Australia, and Spain found that biosolids in the US have significantly higher amounts of PFCA compared to other countries [3]. This difference could be due to the continued use of PFAS precursors in domestic and industrial products in the US or more sensitive methods used to detect PFAS in the reported study [3, 65]. Nevertheless, PFAS concentrations in biosolids in the US have not decreased even after long-chain PFAS use was phased out in the early 2000s [3, 10, 66].

The transformation of PFAS during wastewater treatment processing is of particular concern. A statewide assessment of PFAS in Michigan found that wastewater industrial pretreatment programs were unable to break the C-F bond and instead transformed polyfluorinated precursors to Perfluoroalkyl Acids (PFAAs) which are highly resistant to further degradation, effectively increasing effluent concentrations of total PFAS [14]. The stability of PFAAs, most notably perfluorooctanesulfonic acid (PFOS) and perfluorooctanoic acid (PFOA), has earned PFAS the moniker “forever chemicals” [67, 68].

In addition to being considered a ‘forever chemical’ the fate of PFAS after being land applied is alarming. One of the earliest studies on PFAS in soil following the land application of biosolids found trace levels of perfluorochemicals in soil cores from biosolids-amended soils to depths of 120 cm, suggesting potential movement of these compounds within the soil profile over time [19]. A more recent study [17], investigating the impact of land applying biosolids on the occurrence, concentration, and distribution of PFAS in soils, the vadose zone, and groundwater detected PFAS in all near surface soil samples (< 30 cm below ground surface), in more than 83% of soils between 30 and 90 cm below ground surface, and in the immediately underlying groundwater. PFAS, however, were not detected in adjacent irrigation ditch soil samples where biosolids were not land applied.

**Fig. 1:**
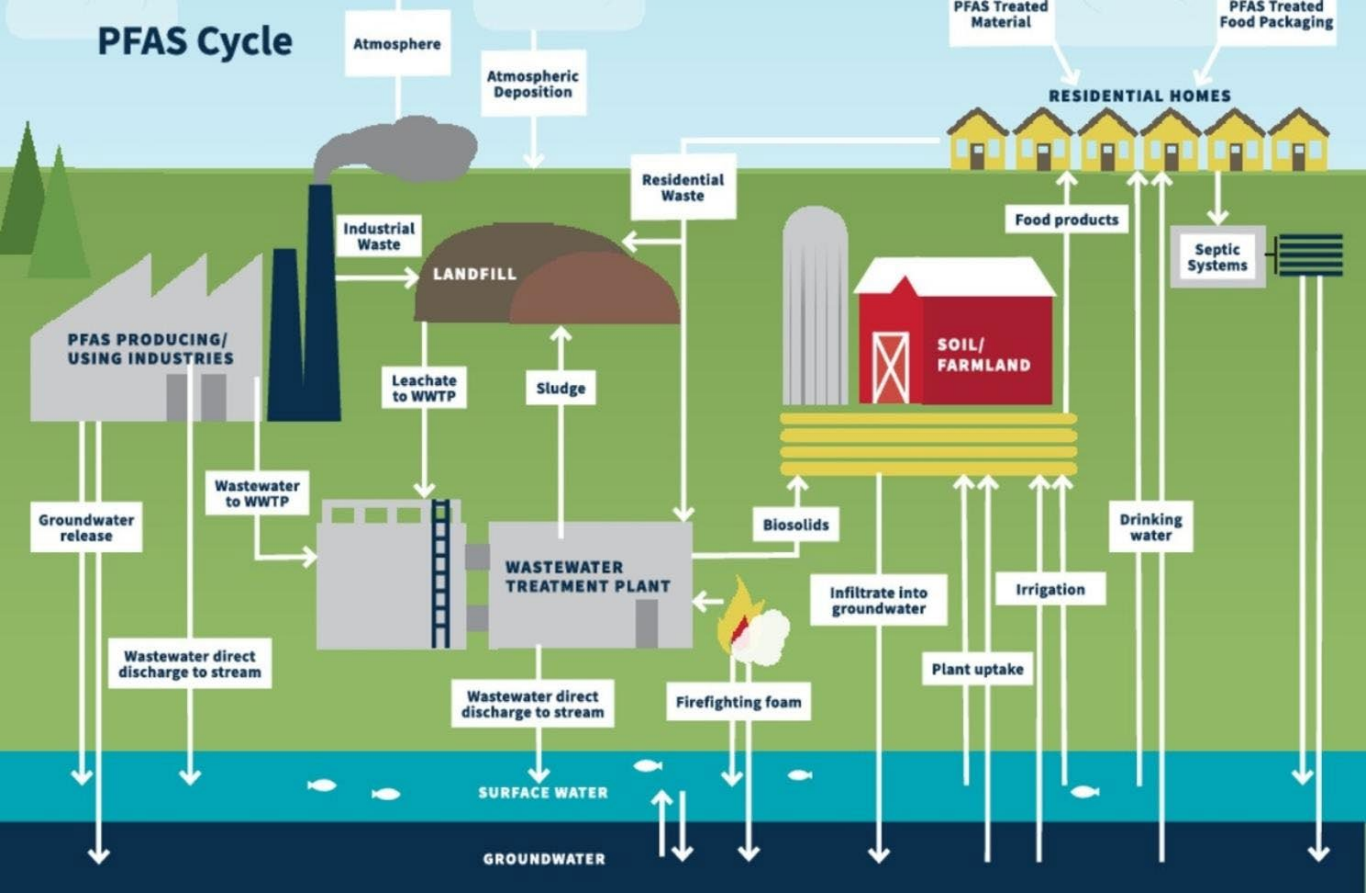
PFAS cycle diagram depicting the movement of PFAS in natural and engineered systems [14]

Like microplastics, PFAS have been detected in the remotest areas on earth [14, 67, 69] and high concentrations of both microplastics and PFAS have even been detected in dust samples [52, 70]. The persistence of PFAS, with a half-life exceeding several decades, leads to complex cycling in the atmosphere, biosphere, geosphere, and hydrosphere (Fig. 1) further raising concerns regarding their ubiquitous distribution into human exposure pathways [3, 58, 71–73].

PFAS can cause adverse health impacts even at ultra-low concentrations, and have been found to bioaccumulate in animals and humans in lung, kidney, liver, brain, and bone tissue [14, 74–82]. According to The California Department of Toxic Substances Control’s recently published chemical profile for PFAS, “*Product – Chemical Profile for Treatments Containing Perfluoroalkyl or Polyfluoroalkyl Substances for Use on Converted Textiles or Leathers” (February 2021 Final Version)*, PFAS exposure is associated with reproductive and developmental, liver and kidney, and immunological effects, as well as tumors in laboratory animals. In addition, inhalation of PFAS can cause acute lung toxicity and inhibit lung surfactant function [3, 83, 84]. The most consistent finding from human epidemiology studies is the increase in serum cholesterol levels among exposed populations. Additionally, there are limited findings correlating exposure to infant birth weights, immune system dysfunction, cancer, and thyroid hormone disruption, and PFAS have also been linked to phytotoxicity, aquatic toxicity, and terrestrial ecotoxicity [84]. The Centers for Disease Control and Prevention (CDC), which monitors Americans’ exposure to PFAS as part of the National Health and Nutrition Examination Survey, has detected PFAS in the blood of all people tested and notes that nearly all humans show evidence of exposure [85]. However, despite this universal exposure and the potential adverse impacts to human health, there is a major data gap in our understanding of the relationship between exposure levels and toxicological outcomes, particularly for PFAS classes other than PFOA and PFOS [86].

The extent of environmental PFAS contamination is also not well quantified; however, based on the usage of biosolids reported by the US EPA and other study estimates, 1760 kg or more of PFAS could be annually deposited onto land directly via land application, from where they could spread into the environment via stormwater runoff, wind, and infiltration into ground water supplies [3, 10, 87].

#### Pharmaceuticals and personal care products

Pharmaceutical and personal care products (PPCPs) are used by consumers for health and cosmetic purposes and by agroindustry to enhance the growth or health of livestock [88–90]. While not all PPCPs are persistent, many are considered “pseudo-persistent” because even though they have high transformation/removal rates this is offset by their continuous use and introduction into the environment [32, 91]. Traditional wastewater treatment plants are unable to effectively remove PPCPs [90, 92] and they can persist through wastewater treatment processes [12, 93–98]. Studies investigating the occurrence and distribution of pharmaceuticals in biosolids following wastewater treatment indicate that pharmaceuticals find their way into the environment mainly through the land spreading of biosolids [32, 99–102].

PPCPs contain chemicals that can disrupt endocrine functions and antibiotics that can lead to acquisition and spread of antibiotic resistance [12, 103]. The potential for reproductive failure caused by endocrine disrupting compounds in aquatic ecosystems (e.g., fathead minnows, zebra fish and white sucker fish) has been extensively documented [12, 104–108]. However, the exposure, toxicity, and bioaccumulation of endocrine disruption compounds in terrestrial organisms has been less well studied. Preliminary evidence has suggested potential risks that are similar to those observed in aquatic species and these compounds have been implicated as potential contributors to diabetes, cancer, fertility decline, and a host of other environmental and public health issues [12]. Ultra-low nanogram per liter (ng/L) concentrations have exhibited impacts to both humans and aquatic organisms, including hormonal interference in fishes, genotoxicity, carcinogenicity in lab animals, endocrine disruption, and immune toxicity [29, 109, 110].

A recent nontargeted analysis using high-resolution mass spectrometry with predictive estrogenic activity modeling was performed on biosolids samples from wastewater treatment plants in California to identify compounds in biosolids that present the most significant environmental barriers to its beneficial use as a soil amendment [23]. The study found that the combination of predictive and in vitro estrogenicity with nontargeted analysis led to confirmation of estrogen-active contaminants in California biosolids and highlighted the importance of evaluating both agonistic and antagonistic responses when evaluating the bioactivity of complex samples. While these findings are compelling, it should be noted that the full spectrum of chemicals that have estrogenic activity and/or can affect the estrogen receptor signaling pathway and subsequent downstream physiological events remains largely unexplored [23].

**Fig. 2:**
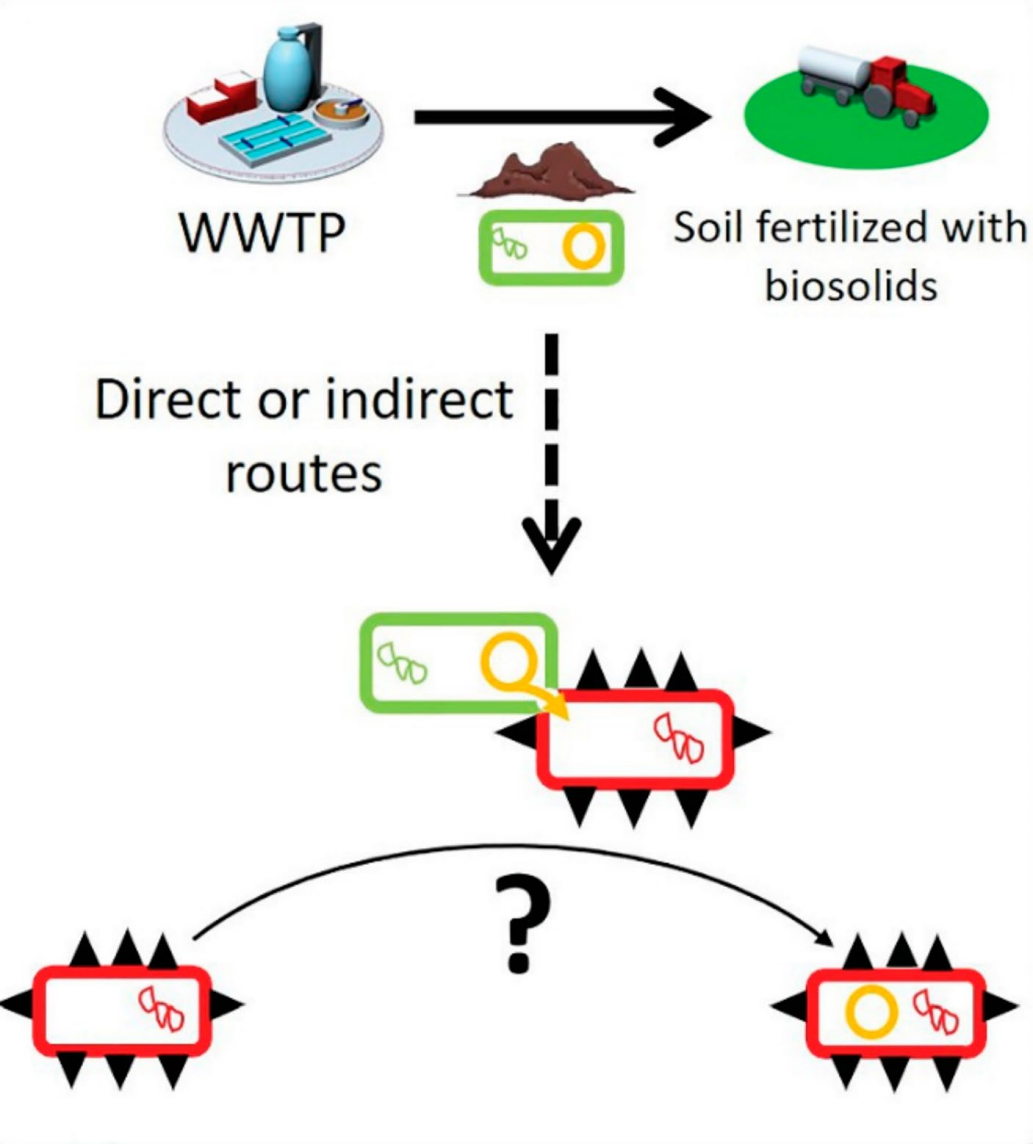
Biosolids from WWTPs used as agricultural soil fertilizer contain bacteria (green rectangles) with resistance plasmids (orange circles). These biosolids can spread the resistance plasmids further, and through direct or indirect routes transfer to human pathogen and commensal bacteria (red rectangles with black spikes) [18]

According to the World Health Organization, the biggest threat to global health, food security, and development is antibiotic resistance, of which PPCPs are a contributor. An increasing number of infections and diseases are becoming difficult to treat, as antibiotics used to treat them become less effective [29, 111, 112]. Our ability to effectively treat infections is hindered by the proliferation of antibiotic resistance genes (ARGs) and antibiotic-resistant bacteria (ARB), which encode various mechanisms conferring drug resistance [15]. A study by Law et al. (2021) found that the spread of ARGs among bacteria is largely driven by the horizontal transfer of mobile genetic elements (MGEs) such as plasmids (Fig. 2) [18]. And they indicate that plasmids are important vectors of horizontal gene transfer and are capable of transferring multiple ARGs simultaneously, providing multidrug resistance to the recipient bacteria in one event. Enrichment of soils with ARGs following the application of biosolids has also been reported [8, 113–115] and given the frequent use of biosolids as fertilizer, their ability to actively transfer resistance genes to pathogens by means of these plasmids is concerning and needs to be further investigated [18]. Wolters et al. [116] also found that disinfectants, heavy metals (including Arsenic as a metalloid), and antibiotics can enhance horizontal gene transfer at sub-inhibitory concentrations. The authors of this study also indicate that use of biosolids as organic fertilizer is contentious – on the one hand, they provide valuable fertilization while on the other they introduce pollutants that likely affect the soil resistome and increase transferability of ARGs, reinforcing the need for further investigation.

A study by Sherburne et al. (2016) also found concentrations of Triclocarban (TCC) and Triclosan (TCS) measured in each trophic level of a terrestrial food web at an agricultural field that had biosolids land applied over a seven-year period. The study investigated a terrestrial food web encompassing biosolids, soil, earthworms (primary consumer), deer mice (Peromyscus maniculatus, secondary consumer), European starlings (Sturnus vulgaris, a secondary consumer of invertebrates), and American kestrels (Falco sparverius, a tertiary consumer of rodents, small birds, and invertebrates) at the biosolids experimental site. The results were compared to the same type of sample results obtained from another agricultural reference site that did not have biosolids land applied and found that antimicrobials were detected in soil as well as in primary (earthworms), secondary (deer mice, starlings), and tertiary (kestrels) consumers. Moreover, concentrations were higher in biosolids (TCC − 1026 − 1472 ng/g wet weight (ww) and TCS − 1114 − 1350 ng/g ww), soil (TCC 14.8 − 27.3 ng/g ww and TCS − 2.7 − 4.4 ng/g ww), deer mice livers (TCC: 12.6 − 33.3 ng/g ww), and starling eggs (TCC: 15.4 − 31.4 ng/g ww) at the experimental site than at the reference site.

Furthermore, a recent study by Hung et al. [15], found that the biosolid samples contained significantly higher levels of selected ARGs than the raw agricultural soils (p < 0.05). Average relative abundances of (intI1, sul1, blaSHV, and ermB) genes were significantly higher in biosolid-amended soils compared to nearby agricultural soils (p < 0.05). A spatial interpolation analysis of relative gene abundances (of intI1, sul1, sul2, and tetW) across the studied area indicated directional trends towards the northwest and southeast directions, highlighting possible airborne spread. Hung et al. concluded that this study brings attention to the need to redefine our antimicrobial standards in soils in terms of public health, in addition to highlighting the importance of considering relatively unstudied transmission routes, such as groundwater and air, when dealing with the current worldwide antibiotic resistance crisis [15].

### Aging, degradation, and synergistic effects of emerging pollutants

The actual fate of organic pollutants in soil is governed by many different factors including soil characteristics, compound properties, and environmental factors such as temperature, precipitation, and the ability of soil microbes to degrade the compound [32, 117]. Emerging pollutants have now also been spread into areas where biosolids have not been land applied because of their persistence during long distance transport and bioaccumulation. For example, land applied biosolids enriched with longer chain PFAS can be adsorbed to microplastics or dust and become airborne [3]. Biosolids can also release fine particles or colloids when subjected to natural drying and freeze thaw cycles, which can carry PFAS to subsurface and ground water [3, 118, 119].

The fate of emerging pollutants in biosolids after land application can vary based on physicochemical properties of the organic compound, the treatment process used to generate the biosolids, and soil properties (e.g., pH and organic carbon), as well as climate [120–125]. Organic compounds present in biosolids, however, can be mobilized during rainfall events following land application, and have been detected in both the dissolved phase, as well as associated with suspended particulates [126, 127]. Gottschall et al. reported the presence of PPCPs in agricultural tile drainage and ground water after application of dewatered biosolids [128]. While Gottschall et al., indicate that the dissipation of many PPCPs in biosolids-amended soils occurs within the first few months after application, some PPCPs, including those in biosolid aggregates incorporated into soil, can still be detected for more than one year following biosolids land application.

Emerging pollutants can also be transformed during degradation into products that have similar biological activity or can have greater toxicity than the parent compound [129, 130]. Macherius et al. reported the formation of triclosan conjugates in carrot cell cultures such that the quantity of conjugates exceeded the amount of parent triclosan by a factor of five [131]. Mordechay et al., reported extensive epoxidation of carbamazepine in the leaves of multiple plant species exposed to the pharmaceutical [132]. More information is needed on the importance of the production of contaminant transformation products after environmental release of these compounds, and the potential for further translocation of pollutants and transformation products beyond plants, especially among nontarget organisms consuming exposed plants [133]. In addition, further study of the role of environmental factors such as rhizosphere microorganisms may play in uptake and transformation of pharmaceuticals in the environment is required [133].

Another factor widely recognized as affecting the bioavailability of organic contaminants in soil is residence time. As soil pollutant contact time increases, pollutant bioavailability and extractability decrease [32, 117]. During aging, pollutants slowly diffuse into the soil matrix via isomorphic dissolution reactions, thus becoming increasingly inaccessible for biodegradation and bioaccumulation [32]. Weathering and aging have been reported to result in decreased toxicity and bioavailability of many soil-applied chemicals [32, 134]. And since long-term aging reactions modify organic contaminants’ availability and toxicity over time, they are important in human and ecological risk assessments and the development of soil quality standards [32]. For instance, sediments can become potentially bioavailable to benthic organisms, and if the level of bioaccumulation is high, they can generate acute and chronic exposure and spread to higher trophic levels [29, 135–137]. Recalcitrant organic contaminants have been found to uptake into plant roots and vegetable crop plants [138–141] and earthworms that can in turn be consumed by other predators in the food chain [142, 143].

**Fig. 3:**
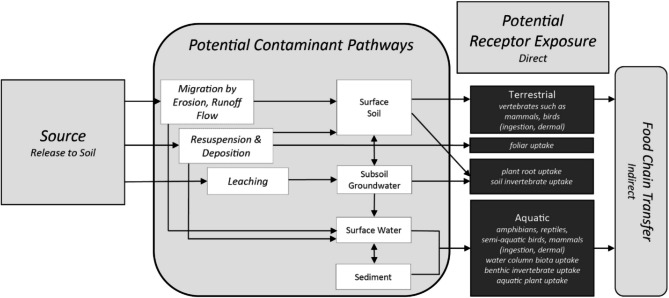
The diagram illustrates relationships between potential exposure pathways and potential ecological receptors after a source releases a stressor to soil [148]

Vasilachi et al. further indicated that if emerging pollutants are in mixtures, the toxic effects can be cumulative and generate synergistic or antagonistic interactions, leading to the so-called “cocktail effect”, so that the difficulty of risk analysis increases [29, 144–146]. Microplastics, for instance, are characterized as being hydrophobic, which makes them inclined to attach to the solid matrix [20, 147]. Studies have also confirmed a high concentration of both and PFAS in dust samples [52, 70]. As a result of these characteristics, the application of biosolids as a fertilizer on agricultural fields may release pollutants such as microplastics and PFAS into the air and pose an inhalation risk because they are more susceptible to suspension by wind than natural soil particles [3]. In this context, the authors indicate that the precautionary principle needs to be applied consistently to ensure a clean and healthy environment for future generations, which is also why further studies on the risks induced by emerging pollutants, due to their specific environmental behavior, toxicity, and impacts on the environment and human health become essential [29]. Figure 3 illustrates the relationships between potential exposure pathways and potential ecological receptors after a source, such as biosolids, releases a stressor to soil.

### Evolution of diagnostic techniques

Although some emerging pollutants and their transformation by-products have existed in the environment for years, their qualitative and quantitative occurrence have been analyzed only recently [27, 29, 101, 149]. New analytical techniques have made it possible to detect and quantify approximately 3000 biologically active chemical compounds in the environment [29, 150–153]. However, efficient and rapid methods for detection in biosolid matrices are lacking.

Detection methods used to count microplastics in biosolids may underestimate or even exclude many microplastics smaller than 10 μm - the fraction that could pose a greater risk for human and animal health [3, 5, 154–156]. Thus, it is critical to develop a simple, rapid method of isolating and quantifying microplastics from environmental samples.

Hutchinson et al. demonstrated through improvements to the analytical method that levels of PFAS in biosolids are significantly higher than historically understood, indicating that the land application of biosolids could result in sensitive environments being exposed to PFAS at levels much higher than previously anticipated [16]. The total oxidizable precursor (TOP) assay, originally published by Houtz and Sedlak [157], has been widely used as an estimate of the total PFAA content of a sample, particularly in wastewater and biosolid matrices; however, it appears this method is failing to adequately digest all the PFAA precursors present in the sample [16]. Still, progress is being made in this arena. The US EPA and the Department of Defense are working to complete a multi-laboratory validation study of a new Method 1633 to test for 40 unique PFAS compounds in wastewater, surface water, ground water, soil, biosolids, sediment, landfill leachate, and fish tissue [158].

Future work is also needed to investigate additional compounds and transformation products that have estrogenic characteristics using non-targeted chemical analysis in conjunction with effects-directed analysis to gain a more comprehensive understanding of endocrine active consumer product chemicals that persist beyond their intended use in consumerism and enter the environment upon ultimate disposal [12].

### Biosolids treatment to remove emerging pollutants

Research and experiments into new technologies, or a combination of technologies are needed to reduce the risk wastewater and biosolids can have on the environment and human health as conventional wastewater treatment plants are, in principle, not designed to remove emerging pollutants [22, 31]. In April of 2020, the United States Environmental Protection Agency (US EPA) formed the PFAS Innovative Treatment Team to explore innovative tools and methods for destroying all the carbon fluorine (C-F) bonds in PFAS-containing waste [159]. The work resulted in improved understanding and advancement of four innovative non-combustion technologies to supplement ongoing EPA research into PFAS treatment. These technologies are electrochemical oxidation (EO), supercritical water oxidation (SCWO), mechanochemical degradation, and gasification and pyrolysis [160].

Berg et al. describes EO as being used to oxidize pollutants by means of passing an electrical current through a solution [159]. They describe that the electronegativity and electron affinity of fluorine allows the C-F bond to be broken and the fluorine atoms reduced when a high overpotential is applied to a solution. Their review of SCWO indicates that preliminary studies on biosolids treatment have shown strong reductions in PFOS and PFOA levels in the processed effluent. They also indicate that sufficiently dewatered sludge, or wet sludge dried by addition of co-milling agents, would be applicable to treatment in a mechanochemical degradation system. However, they indicate no such tests with PFAS have been identified in the literature. They further indicate that PFAS testing on a biosolids pyrolysis system was repeated in a test commissioned by the PFAS Innovative Treatment Team confirming high levels of degradation of the target PFAS compounds analyzed in the feed. Research to evaluate these promising technologies for PFAS destruction are ongoing.

Chen et al. indicates that hydrothermal processing (HTP), including hydrothermal carbonization (HTC) and hydrothermal liquefaction (HTL), is a promising method to sustainably manage biosolids because it can convert it into useful products while mitigating the environmental risks of biosolids simultaneously [161]. They describe that products from HTP are normally biocrude oil, aqueous products (containing fertilizer precursors), and hydrochar. The authors also indicate that HTC has been extensively used for phosphorus recovery, while HTL can effectively remove constituents of emerging concern. They go on to indicate that energy analysis of HTP indicates that HTP has a 11-fold higher energy recovery than landfilling [162].

Low temperature mineralization of perfluorocarboxylic acids is also being experimented with. Trang et al. found that perfluoroalkyl carboxylic acids (PFCAs) could be mineralized through a sodium hydroxide-mediated defluorination pathway [163]. Their study found that PFCA decarboxylation in polar aprotic solvents produced reactive perfluoroalkyl ion intermediates that degraded to fluoride ions (78 to ~ 100%) within 24 h. The study also indicates that degradation was observed for branched perfluoroalkyl ether carboxylic acids. They surmised that this may then inform the development of engineered PFAS degradation processes and facilitate expanding this reactivity mode to PFAS with other polar head groups.

For perspective, and as described by Vasilachi et al., source reduction and substitution of emerging pollutants with products having lower toxicity and easier removal from water have played an important role in reducing the impact of emerging pollutants on the environment and human health [29]. Amending biosolids with biochar or wood chips has been demonstrated to significantly enhance the degradation and/or retention (sorption) of target total and(or) leachable pharmaceuticals [164]. However, one should keep in mind that the solution to pollution should not always be dilution, so efforts to treat biosolids to remove contaminants of potential risk to health and the environment should continue to be pursued, particularly due to the demonstrated biopersistence of many of these synthetic organic chemicals. Related to PFAS, another study of commercial biosolids from the U.S. and Canada found that while thermal hydrolysis had no apparent effect on the PFAA concentration, heat treatment and composting increased PFAA concentrations (especially PFHxA) via the degradation of precursors [165]. Only blending with PFAS-free material decreased the concentration of PFAAs in the commercial biosolids, by diluting it. With millions of tons of biosolids being land applied annually, dilution cannot be a sustainable or a long-term treatment solution to removing contaminants that persist and bioaccumulate in the environment.

The California Department of Toxic Substances Control chemical profile for PFAS, “*Product – Chemical Profile for Treatments Containing Perfluoroalkyl or Polyfluoroalkyl Substances for Use on Converted Textiles or Leathers” (February 2021 Final Version)* indicates that intentional or accidental combustion of PFAS forms hazardous chemicals [84]. For instance, the combustion of various fluorinated polymers can result in emissions of C3-C14 PFCAs, ozone-depleting substances such as chlorofluorocarbons, and greenhouse gases such as fluorocarbons when fluoropolymers are combusted at temperatures representative of municipal incinerators [166]. During incineration at temperatures above 450 °C, Polytetrafluoroethylene (PTFE) (aka Teflon) also forms additional hazardous substances including the ultra-short-chain PFAA tetrafluoroacetic acid and hydrofluoric acid (HF) [166, 167]. Moreover, an industry-sponsored study in a rotary kiln test facility simulating municipal incinerators found that PTFE polymer pellets begin to decompose at around 500˚C, and by approximately 650˚C they completely convert to HF gas and F-containing ash, with no significant PFAA emissions [168]. Other authors have reported that at lower temperatures, as could occur during accidental landfill fires, fluoropolymers such as PTFE can break down into PFCAs, including PFOA [169, 170].

In this regard, future technologies need to be both effective and environmentally-friendly treatments, capable of removing the widest possible spectrum of emerging pollutants, with low energy consumption and capital expenditures. And the efficiency of the treatment must be adjustable to emerging pollutants concentrations in an aquatic environment to make it possible to recover the treated water [29].

### Monitoring and regulations

The lack of standards and regulations for the emerging pollutants discussed in this review are due, in part, to lack of available data on the effects of chronic exposure on human health. This underscores the need for complete epidemiological and toxicological studies, in addition to the development of better treatment technologies, and standardized diagnostic testing methods for monitoring emerging pollutants in biosolids. Additionally, systematic approaches are needed to identify and prioritize pollutants of emerging concern [10, 171]. A complete ban on land application would not only place a heavy burden on public municipalities but could also lead to unintended consequences [172].

Recently, two states in the US have adopted regulations relating to the presence of PFAS substances in biosolids. In 2021 under an interim strategy, Michigan began prohibiting the land application of industrially impacted biosolids containing more than 150 parts per billion (ppb) of PFOS and requires testing of biosolids prior to land application. On April 15, 2022, the Maine state House and Senate both passed a bill (LD 1911) that would ban the use of biosolids that contain PFAS in land applications, unless it can be shown that the biosolids are PFAS free.

On the national level, in a move toward regulation of PFAS, EPA Administrator Michael S. Regan announced the Agency’s PFAS Strategic Roadmap on October 18, 2021. The roadmap charts an approach to addressing PFAS with EPA’s Commitments to Action steps that take place between 2021 and 2024. In addition, the EPA’s Office of Water developed a Biosolids Chemical Risk Assessment and Biosolids Screening Tool (BST) with an accompanying User Guide to identify pollutants, pathways, and receptors of greatest interest and to inform decisions regarding the need for refined risk assessment of land-applied biosolids. In late 2021, the US EPA also selected a Science Advisory Board to review and provide input on the overall risk assessment approach and on the scientific credibility and usability of the BST. But these actions have not yet translated into protective standards for public health with respect to biosolids land application.

## Discussion

From a regulatory standpoint, there is an urgent and critical need to modernize environmental health standards that pertain to the land application of biosolids. Current US EPA regulatory standards have not been updated since 1993 and do not take the emerging contaminants described in this review into account. The revision of these standards should be risk-based, prioritizing emerging pollutants that are persistent and can bioaccumulate. Support should be provided for the development of novel technologies to better treat biosolids to remove the contaminants of concern before they are land applied and novel methods for recycling or reuse of these pollutants should be explored. However, these changes require political will and collaboration among state and federal agencies to prioritize new policies and regulations.

Ongoing development of analytical methodologies for identifying emerging contaminants in soil, water, waste, and other media should be a priority of relevant regulatory agencies. Of note, Hutchinson et al. indicate that a standardized analytical methodology is needed to protect environmental assets from PFAS contamination from land-applied biosolids [16]. Inconsistencies between current testing methods used to detect and measure emerging pollutants, likely underestimate concentrations of these pollutants in receiving matrices, especially where published quantification methods are adapted to biosolids but not the receiving matrix. At the time of this writing, the US EPA is developing a new standardized testing methodology; however, the authors’ conclusions remain relevant as prior studies have not captured a complete picture of the extent and impact of contamination.

More research is also needed to assess the long-term human health risks from exposure to emerging pollutants. The ecological risks of microplastics in agricultural soils urgently need to be assessed with respect to animals, plants, and microorganisms inhabiting soils and humans involved via the food web. Additionally, future studies should measure microplastics and associated PFAS in dust from biosolids and quantify exposure risks via inhalation. Studies evaluating the factors that contribute to the fate of emerging pollutants must be conducted to better understand exposure routes and the risks of surface and ground water contamination with these pollutants.

Lastly, effective communication to the public on the significance of pharmaceutical ingestion and personal care product use and the resulting environmental effects due to runoff from agricultural land may help to put pressure on decision-makers and create an awareness of unwarranted excessive use of these products [32]. This also presents an opportunity to promote use of more sustainable products to reduce the volume of contaminants that are released to the environment via wastewater infrastructure pathways.

## Conclusion

Humans are being continuously exposed to the emerging pollutants described in this review and a concerted effort should be made to mitigate these exposures and risks among the general public, policymakers, wastewater treatment plant operators, and farmers, in terms of raising awareness, controlling the sources, and establishing a reasonable regulatory risk level for biosolids reuse. As a society we must also promote the use of more environmentally sustainable products that can be flushed into the environment.

Research indicates that biosolids contain a complex mixture of contaminants, and investment in effective treatment and diagnostic technologies are essential for detecting and reducing the presence of contaminants so the benefits of biosolids related to carbon sequestration and soil health can be fully realized. We should reconsider the continued land application of biosolids in this seemingly endless loop of spreading contaminants into our environment, and we should most certainly not continue to do it in the name of recycling, climate change, and soil health. Instead, efforts need to be pursued to work within the current system to better communicate and act upon the human health risks in order to achieve desired public health outcomes. Significant funding and support for upgrading wastewater treatment infrastructure are also needed to address these issues of today in order to better prepare for a safer tomorrow.

A final note to ponder as we consider this issue. Biosolids, in their current form, have often been referred to as an organic waste to be recovered and recycled. But given the presence of contaminants that originate from both domestic and industrial wastewater sources, is that really the appropriate designation in law or regulation? As a society, if we fail to take definite policy actions to modernize environmental standards that pertain to the land application of biosolids, and continue to land apply layer upon layer of these complex mixtures of pollutants to our soil without adequate public health protections in place, and without regard to the long-term environmental consequences, we may potentially cause irreversible damage to the very soils we use to grow our food and to our surface and ground water that sustain life.

### Electronic supplementary material

Below is the link to the electronic supplementary material.

**Supplemental Fig. 1.** PRISMA Flow Diagram

## Data Availability

Not applicable. All literature cited is available at the reference provided.
