# Supplementary figures and images for: Emerging environmental health risks associated with the land application of biosolids: a scoping review
Source: Environ Health. 2023 Aug 21;22:57. doi: 10.1186/s12940-023-01008-4 (PMC10440945; doi:10.1186/s12940-023-01008-4)

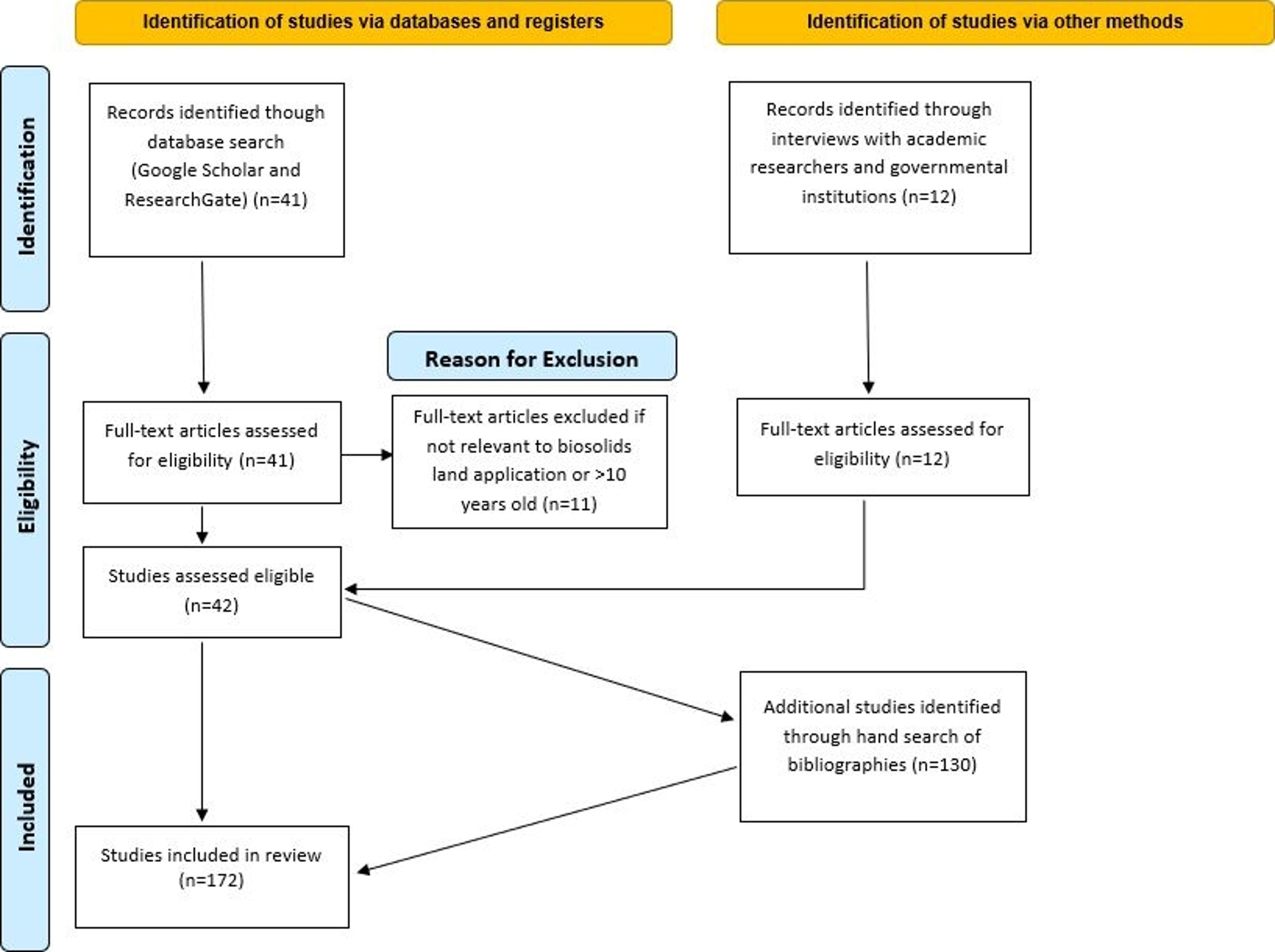

Supplement: Supplementary file 1 — Supplemental Fig. 1. PRISMA Flow Diagram [file 12940_2023_1008_MOESM1_ESM.tif]
